# Editorial: Stem cell regeneration strategies in treating kidney diseases: From mechanisms to therapeutics

**DOI:** 10.3389/fcell.2023.1140819

**Published:** 2023-02-09

**Authors:** Chee-Yin Wong, Angela Min Hwei Ng, Yang-Mooi Lim, Soon-Keng Cheong

**Affiliations:** ^1^ M. Kandiah Faculty of Medicine and Health Sciences, Universiti Tunku Abdul Rahman, Kajang, Selangor, Malaysia; ^2^ Centre for Tissue Engineering and Regenerative Medicine, Faculty of Medicine, Universiti Kebangsaan Malaysia, Kuala Lumpur, Malaysia

**Keywords:** stem cells, kidney disease, kidney regeneration, cell therapy, regenerative medicine, extracellular vesicles, mechanism

World Health Organization estimated 5 to 10 million people worldwide die from the lack of access to critical treatments for kidney disease annually (Luyckx et al., 2018). Kidney disease remains a global cause of global morbidity and mortality. By 2040, chronic kidney disease is projected to be the fifth leading cause of mortality globally (Luyckx et al., 2021). Despite significant advances, currently available drugs are only effective in delaying disease progression. Reversal of kidney damage or kidney regeneration using pharmacotherapy remains futile. Moreover, the current therapeutic repertoire to prolong the lifespan of the end-stage kidney disease patients is limited to kidney replacement therapy, such as dialysis and organ transplantation. Dialysis is not an ideal solution due to the high medical cost involved and that this therapy compromised the patients’ quality of life. Meanwhile, the severe shortage of organ donors and potential organ rejection risks limit the practice of kidney transplantation. It is therefore crucial to discover other types of treatment that can improve the kidney patients’ quality of life while also possibly cure, reverse, or alleviate the kidney disease. Stem cells have the potential to alter the landscape of kidney disease in the form of regenerative medicine. There are increasingly stronger evidence from experimental kidney disease studies that stem cell therapy can treat the impaired kidney and attenuate kidney damage while improving the structure and function of glomerular and tubular components.

Based on the topic of stem cells and kidney disease, this compilation includes one original research article and three review articles to explore the potential strategies, therapeutic effects, and mechanisms of stem cell-based therapy, including its derivatives, to treat kidney diseases as well as to provide new insights.

The review by Tsuji et al. discussed and summarized the potential strategies for kidney regeneration with stem cells in two directions: *de novo* whole kidney fabrication with stem cells and stem cell therapy. *De novo* whole kidney fabrication is developed on the concept of “rebuild”, i.e., constructing the whole organ for transplantation, while stem cell therapy is based on the concept of “repair”, i.e., inducing native kidney repair system.

To date, most stem cell clinical trials for kidney disease used mesenchymal stem cells (MSCs) in their approach (Wong, 2021). Wang et al. summarized the current usage of MSCs in chronic kidney disease at pre-clinical or clinical studies. The review by Wang et al. also discussed the challenges of MSCs therapy in translational research for chronic kidney disease.

In recent years, the possibility of administering extracellular vesicles (EVs) have gained much interest. In addition to its ability to maintain or mimic stem cells’ effects, the advantage of these cell-free agents bypassing most of the safety concerns related to stem cell-based therapy, have paved the use of EVs in clinical trials (Yin et al., 2020). Hun et al. performed a bibliometric analysis to provide an overview on the current trends in EVs research for kidney-related diseases. They found this research area to be growing rapidly with its domain likely to expand in the next decade. Liao et al. further explained the advantages of EVs in comparison to cellular-based therapy and summarized the mechanisms that were discovered to date.

In summary, this research topic provides a platform to exhibit a number of comprehensive original and review articles to highlight the promising potential of stem cell-based therapy, including its derivatives, to treat kidney diseases and to bring us closer to real-life regeneration therapeutic interventions in the near future.
